# A systematic review of stakeholder views of selection methods for medical schools admission

**DOI:** 10.1186/s12909-018-1235-x

**Published:** 2018-06-15

**Authors:** M. E. Kelly, F. Patterson, S. O’Flynn, J. Mulligan, A. W. Murphy

**Affiliations:** 1 Discipline of General Practice, Clinical Science Institute, National University of Ireland, Galway, Ireland; 2 University College Cork, Cork, Ireland; 3 Work Psychology Group, Derby, UK

**Keywords:** Stakeholders, Views, Organisational justice, Medical schools

## Abstract

**Background:**

The purpose of this paper is to systematically review the literature with respect to stakeholder views of selection methods for medical school admissions.

**Methods:**

An electronic search of nine databases was conducted between January 2000–July 2014. Two reviewers independently assessed all titles (*n* = 1017) and retained abstracts (*n* = 233) for relevance. Methodological quality of quantitative papers was assessed using the MERSQI instrument. The overall quality of evidence in this field was low. Evidence was synthesised in a narrative review.

**Results:**

**Applicants** support interviews, and multiple mini interviews (MMIs). There is emerging evidence that situational judgement tests (SJTs) and selection centres (SCs) are also well regarded, but aptitude tests less so. **Selectors** endorse the use of interviews in general and in particular MMIs judging them to be fair, relevant and appropriate, with emerging evidence of similarly positive reactions to SCs. Aptitude tests and academic records were valued in decisions of whom to call to interview. **Medical students** prefer interviews based selection to cognitive aptitude tests. They are unconvinced about the transparency and veracity of written applications. Perceptions of organisational justice, which describe views of fairness in organisational processes, appear to be highly influential on stakeholders’ views of the acceptability of selection methods. In particular procedural justice (perceived fairness of selection tools in terms of job relevance and characteristics of the test) and distributive justice (perceived fairness of selection outcomes in terms of equal opportunity and equity), appear to be important considerations when deciding on acceptability of selection methods. There were significant gaps with respect to both key stakeholder groups and the range of selection tools assessed.

**Conclusions:**

Notwithstanding the observed limitations in the quality of research in this field, there appears to be broad concordance of views on the various selection methods, across the diverse stakeholders groups. This review highlights the need for better standards, more appropriate methodologies and for broadening the scope of stakeholder research.

**Electronic supplementary material:**

The online version of this article (10.1186/s12909-018-1235-x) contains supplementary material, which is available to authorized users.

## Background

Medicine is a highly popular career choice internationally. For example, each year in the UK alone there are over 19,000 applicants to medicine and approximately 42,000 in the USA [1]. Likewise, selection to internship and residency training programmes is very competitive and these high stakes assessments determine which graduates ultimately work in the various specialities. As attrition rates in medical education are very low and most students graduate, the composition and calibre of the future medical workforce is significantly dependent on the methods used to select medical students [2–4]. Hence medical student selection is a topic of considerable public interest with numerous stakeholder groups. These include applicants and potential applicants; selectors such as medical school admissions committees; medical students; the medical profession; school career guidance teachers and society. Arguably, the most important stakeholders are patients. Best practice in the design, development and continued use of selection methods should be an iterative process informed by regular feedback from stakeholders [5]. The term political validity captures the centrality of stakeholder views and, is defined as “the *extent to which various stakeholders and stakeholder groups consider the tool(s) to be appropriate and acceptable for use in selection*” [6, 7]. Political validity is recognised as an important consideration in widening access to medical schools [8, 9]. Elsewhere it has been argued that political validity is to some extent informed and influenced by evidence for the construct validity of selection tools [10]. According to Kane construct validity *“is a property of the proposed interpretations and uses of the test scores*” [11]. Five sources of evidence to support test interpretation are recommended: test content; relationship to other variables, response process, internal structure and consequences of testing [12]. It is beyond the scope of this paper to provide an in-depth definition of these and the reader is directed to the most recent edition of Standards for Educational and Psychological Testing, for further information [13]. However it is likely that different sources of evidence, exert varying degrees of influence on stakeholders’ opinions, and this may differ depending on the stakeholder group in question.

Understanding stakeholder perceptions is important for a number of other reasons. Selection methods that are *perceived* as unfair may deter potential medical students from applying which would be considered a profoundly negative consequential effect [9]. Under-representation of lower socio-economic and minority groups in medicine is multifactorial but arguably these groups are particularly vulnerable to the consequences of negative perceptions regarding selection [14]. Additionally, in some situations, there appears to be a trade-off between stakeholder views and other criteria used to evaluate the appropriateness of selection tools, such as predictive validity and reliability. For example personal statements, letters of reference and traditional interviews continue to enjoy widespread use, despite evidence of limited predictive validity and susceptibility to bias [15–19]. It has been argued that this can in part be explained by these tools serving some other political agenda for which they achieve stakeholder acceptance and approval [9]. It is crucial therefore that stakeholder views are explored, understood and communicated effectively in order to increase the likelihood that selection tools can be developed that can meet with stakeholder approval whilst also satisfying the other important psychometric criteria. Finally a thorough understanding of the basis for stakeholders’ views will better enable selectors to explain the rationale supporting some, perhaps less popular, but more psychometrically robust selection tools.

### Stakeholder views: a theoretical framework

Over the past fifty years, organisational justice theories have been developed to describe perceptions of fairness in organisational processes, including selection [20–22]. Patterson et al. and Kelly have established that organisational justice theories are relevant to selection in medicine and that they can be used to provide deeper insights into and appreciation of the views of stakeholders [7, 23].

These justice theories can be categorised as distributive, procedural and interactional- see glossary [24]. In the context of selection **distributive justice** relates to the fairness of selection outcomes - such as medical school places, in terms of equal opportunity and equity [7]. From a distributive justice perspective selection is viewed to be fair when everyone receives the same opportunities [25]. **Procedural justice** in selection is concerned with the perceived fairness of the selection tool in terms of job relevance and characteristics of the test [7]. From a procedural justice perspective selection is viewed more positively when the methods are connected with the job and when the purpose of the method is explained [22, 26]. While the **interactional justice** of selection methods refers to how applicants are met during the selection process and includes the information applicants are given as well as the manner in which it is conveyed [27, 28]. The fairness of the communication is a very influential determinant of how interactional justice is perceived [28, 29].

Despite their significance, to our knowledge, there has been no review that draws together the views of stakeholders when considering the appropriateness of various selection methodologies. Therefore this review is necessary and timely, as important questions remain to be answered. This study aims to (i) systematically review the literature with respect to stakeholder views of selection methods for medical school admissions; (ii) relate the findings to organisational justice theories and (iii) identify priority areas for future research.

## Methods

There was no published review protocol.

### Search strategy

Data searching and subsequent critical review of identified articles was informed by best evidence medical education guidelines [30–33]. The search strategy was developed in collaboration with a research librarian (JM). Nine electronic databases were searched: PubMed, EMBASE, SCOPUS, OVID Medline, PsycINFO, Web of Science, ERIC, British Education Index and Australian Education Index. Relevant papers were identified using search terms (including synonyms) for each of the four concepts “stakeholder”, “views”, “selection” and “medical school”. Terms were mapped to MESH terms or the appropriate term from the controlled thesaurus of the various databases. In addition, text word searches were used for key words. See Additional file 1 for sample search.

For the purposes of this review “Stakeholders” were defined as those who are affected by or can affect recruitment processes [34]. The search terms for stakeholder were deliberately cast widely to encompass as many stakeholder groups as possible. “View” was defined as an opinion or attitude. “Selection” was taken to mean any admission test or entrance assessment process that a medical school applicant would have to go through in order to be offered a place. “Medical school” was taken to include both graduate and undergraduate schools. Additionally, as there is significant overlap between some methods used for selection to medical school and selection to higher professional training (for example Multiple Mini Interviews (MMIs) and Situational Judgement Tests (SJTs) are increasingly used in both settings) this search was widened to include internship and residency. Within each concept, terms were joined using the Boolean operator “OR”. The four searches were then combined with the operator “AND”. Language or type of publication restrictions were not applied during the searching phase. The reference lists of papers included in the review were hand searched for additional relevant publications. Two experts in the field were contacted for any additional records or unpublished work. Further grey literature searching was facilitated by searching for conference publications and networking with researchers in the field which provided access to unpublished reports, doctoral theses work and abstracts.

The inclusion criteria were: (a) Studies published between January 2000 and July 2014 (this time frame was chosen as many of the selection methods in current use were neither available nor widely used prior to 2000) (b) Studies evaluating selection to medical school or studies evaluating selection to residency and internship programmes which described selection processes relevant to selection to medical school (for example- studies focussing on the residency match rank process were not included) (c) Studies which reported the views of at least one stakeholder group established by means of quantitative, qualitative or mixed methods research. The exclusion criteria were: (a) Reviews or articles which were not original studies (b) Papers for which an English language translation was not available on contacting the authors. As this was a systematic review which did not involve any original stakeholder data, ethical approval was not required.

## Results

### Study selection and data extraction

**Fig. 1:**
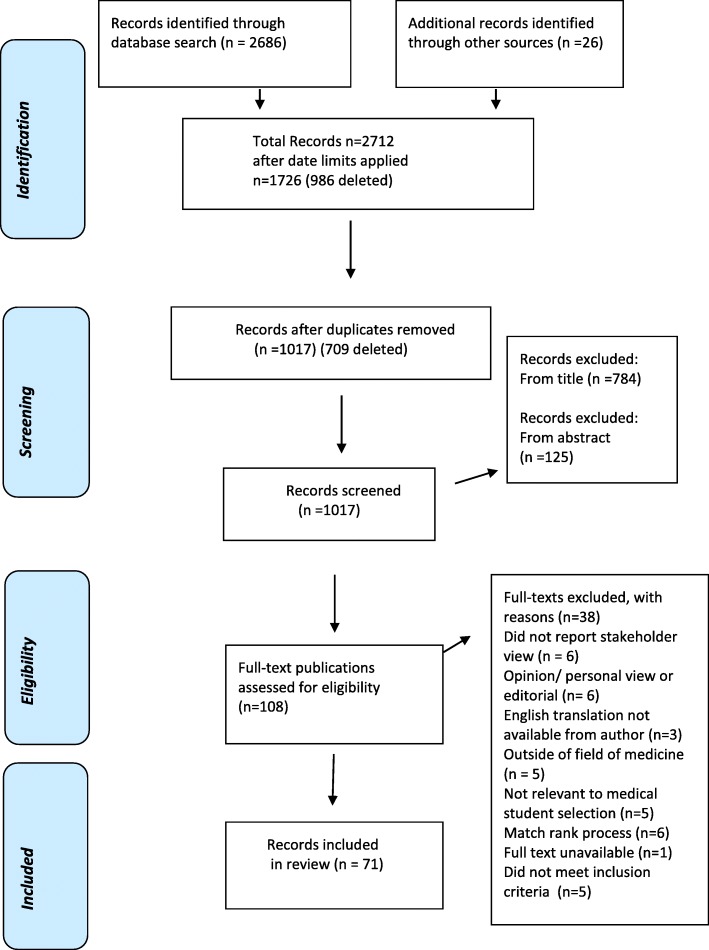
Study Search Strategy and Review Process

Figure 1 illustrates the steps from initial identification of records, to identifying those included and excluded. Records were retrieved from the electronic search as follows: all records identified in the electronic database search (total *n* = 2686) and by the additional means described above (*n* = 26) were transferred to EndNote database, duplicates were removed (by automatic deduplication and manual check) and the remaining records were inspected (*n* = 1017).

Two reviewers (MK and AWM) independently assessed all titles for relevance (*n* = 1017). Where disagreement arose the record was included for review of abstract. These reviewers also independently screened abstracts of all retained records (*n* = 233) to identify those to be assessed on full text, with 95.71% agreement. This left a total of 108 records which were read in full by three reviewers (MK, AWM, SO’F) and independently assessed for eligibility to be included in the full review. Disagreement was managed by consensus in consultation with another author (FP). Subsequently 70 records were included for full review and 38 excluded. Figure 1 indicates the reasons for exclusion.

The following data were collected from each eligible record and collated in a data extraction form: author, publication year, type of publication, principal study aim, location and setting, study design, medical selection tool used, stakeholder characteristics (including identification of stakeholder group, sample size, response rate, gender, age, socioeconomic group, background if provided), data collection method and overall findings.

### Quality assessment strategy

Quality criteria of quantitative records was assessed using the Medical Education Research Study Quality Instrument (MERSQI), a commonly used validated ten-item checklist for rating the methodological quality of medical education research papers [35, 36]. This instrument has six domains (study design, sampling, type of data, validity of evaluation instrument, data analysis and outcomes). As a quality assurance step, at the outset, a sample of five records were independently scored using the MERSQI instrument by MK and AM. The scoring was discussed and debated and consensus was reached as to the interpretation of the scoring grid. For consistency one author (MK) then applied the MERSQI instrument to the retained records. For studies with multiple aims, for example assessing predictive validity and stakeholders’ views, the MERSQI rating was applied to the portion of the study that assessed stakeholder views, as this is the subject of this systematic review. It was not appropriate to use MERSQI to assess qualitative studies, and with respect to mixed methods studies, the score refers to the quantitative strand only.

### Quality assessment and evidence synthesis

The MERSQI ratings for the included records ranged from 3 to 10.5, out of a total possible score of 18. The mean MERSQI score was 7.2 and the median 7.5 (total MERSQI scores, for all included records, are presented in Table 1. Additional file 2 presents the completed MERSQI scoring matrix for all records). By comparison, a review of over 200 published peer review medical education papers determined that the mean MERSQI of published papers was 9.95 (range 5–16) [36]. This indicates that the overall quality of the retained records was generally low, reflecting the standard of currently available literature on stakeholder views. (See Study Limitations in the Discussion section). MERSQI scores were used to compare quality and were not used for the purpose of excluding records from this review. Due to the heterogeneity of studies, and the wide variety of selection methods and evaluative measures used, it was not possible to pool results statistically. Therefore the evidence is synthesised into a narrative review.

**Table 1:** Summary of the Research Evidence of Stakeholder Views of Selection to Medicine

Author /Year/ Type of article	Location / Setting	Study Design / Aim	Selection tool(s)	Stakeholder/ number / response rate(RR)	Data Collection method	Outcome variable relevant to this review	Overall findings relevant to this review	MERSQI Quality
Adams 2009 ^a^ [52] Research paper	USA Medical Schools	Quantitative	Academic record (compared acceptability of different educational settings)	Medical school administrators *N* = 58, RR 49%	Postal questionnaire survey, with 3 hypothetical scenarios to rank, checkboxes and open questions.	Offer of interviews, based on applications that were identical except for the institution where the applicant received their academic qualification.	University academic record perceived as preferable to both community college courses and online courses by selector administrators, in deciding offers of interviews, citing concerns about quality and rigor.	7
Agrawal et al. 2005^a^ [53] Research paper	USA Medical Schools	Quantitative	Aptitude Test / MCAT/ Academic Record GPA / Other factors	Deans of Student Affairs *N* = 86, RR 59%	Postal questionnaire, based on extensive literature review & piloted.	Percentage responses	MCAT (90%), GPA (60%), lack of financial aid (48%), lack of role model (77%) seen as barriers to under-represented and minority applicants by deans	8.5
Brown & Griffin 2012 ^a^ [54] Abstract	Australia 1 medical school	Quantitative	Academic record/ UMAT or GAMSAT and interviews	Mixed stakeholder group- comprising applicants medical students patients and doctors. *N* = 938 RR = UTD	Questionnaire	Collated views of perceived validity, familiarity, overall confidence in selection	Confidence in selection methods low for all groups, even lower in medical professionals. Cognitive ability tests least valid/ interviews most valid by this mixed stakeholder group.	6
Brownell et al. 2007^a^ [55] Research paper	Canada 1 Medical School	Quantitative	MMI	Applicants *N* = 277 RR 98.5% Interviewers *N* = 74, RR 91%	Questionnaire	Mean ratings and standard deviations of 5 point Likert scale responses (1 = strongly disagree, 5 strongly agree)	MMIs acceptable to both. Applicants free from gender (4.7) & cultural bias (4.6), adequate time (3.6) stressful (2.9). Interviewers had adequate orientation (4.4) & preparation (4.6), scoring sheet allowed differentiation (4), adequate time (4.1) MMI fair (4.3).	8
Campagna-Vaillancourtet al 2013^a^ [45] Research paper	Canada Postgraduate residency programme – Ears, Nose Throat, Head & Neck	Embedded mixed methods	MMI	Applicants *N* = 45, RR 100% Interviewers *N* = 18, RR 100%	Questionnaire survey with open ended qualitative comments	Mean ratings and standard deviations of 7 point Likert scale responses (1 = strongly disagree, 7 = strongly agree)	MMIs acceptable to both. Applicants: MMI free from gender (6.2), cultural (6.2), age bias (6.3); adequate time (6.1), preferred to TI (5.3), Interviewers: better than TI (5.3) adequate preparation (5.2), scoring allowed differentiation (4.7), adequate time (5.4) MMI fair (6).	8.5
Christakis et al. 2010 [56] Research paper	Canada 1 Postgraduate residency – ophthalmology	Quantitative	Interviews day	Applicants from two years (2000, 2005). *N* = 26, RR 79%	Questionnaire – survey, with Likert scale responses & some overlap of questions between the two surveys.	Frequency of responses, from both years merged.	14/14 applicants felt the interview day was valuable, 14/14 appropriate length, 14/14 adequate opportunity to express own ideas and opinions. 20/26 felt the interview day affected their opinion of the programme positively and increased their likelihood of accepting a place.	7.5
Cleland et al. 2011^a^ [46] Research paper	Scotland 5 Undergraduate Medical Schools	Mixed Methods	Aptitude Test/ UKCAT	1st Yr Medical students Qualitative strand *N* = 28 Quantitative strand *N* = 883, RR = 88%	Focus groups & Questionnaire survey (4 items relevant to this review)	Framework analysis & Percentage responses	Medical students viewed UKCAT poorly, lacking face validity, poor predictive validity, coachable, discriminatory against less affluent applicants. Only 20% agreed that UKCAT was useful.	9
Daram et al. 2014 [57] Research paper	USA 1 Medical Fellowship – Gastroenterology	Quantitative	Web based video conferencing interviews (WBVC)	Applicants *N* = 16, RR = 100%	Questionnaire	Percentage responses	13 candidates (81%) felt WBVC interview met or exceeded expectations, 87% thought WBVC should be an option in fellowship interviews. 25% felt that WBVC was equivalent to or better than their traditional interview experience.	6
Dennehy et al. 2013^a^ [58] Research paper	Ireland Medical School Entry – Survey conducted in one geographical area	Quantitative	Aptitude Test- HPAT-Ireland/Academic record	General practitioners (GPs) *N* = 122, RR = 79%	27 item Questionnaire survey	Percentage responses	GPs supported the use of aptitude tests in principle (69.7%) & academic record (96.7%) but 30% unhappy with reforms introducing aptitude tests. Concerns expressed re socio-economic bias of academic record (71%) & HPAT-Ireland (66%)	7.5
Dhar et al. 2012^a^ [59] Research paper	New Zealand 2 Undergraduate Medical Schools	Quantitative	Aptitude Test / UMAT	Medical students *N* = 1325, RR 65%	35 Item Questionnaire survey- closed questions & 4 or 5 point Likert scales.	Binary logistic regression & percentage responses	56% of students thought UMAT was not important for selection, 67% was not fair, 81% stressful, 54% felt it assessed non cognitive attributes not really or not at all.	8
Dore et al. 2010^a^ [60] Research paper	Canada Post graduate programmes in Obstetrics, Paediatrics, Internal Medicine	Quantitative	MMI	Applicants- *N* = 484 RR = UTD MMI Assessors – *N* = UTD, RR = UTD	Questionnaire survey	Percentage responses to satisfaction ratings	Applicants: 88% believed they could positively portray themselves & 70% felt they had adequate time. Assessors: 90% felt they had a reasonable portrayal of candidate’s abilities 74% felt MMI better than TI	7.5
Dowell et al. 2012 [61] Research paper	Scotland 1 Undergraduate Medical School	Quantitative	MMI	Applicants *N* = 324, RR 75% Interviewers – *N* = 116, RR = 58%	Online Questionnaire	Percentage responses	94% applicants thought MMIs fair, 33% more stressful than TI, 74% preferred MMI to TI 90% of interviewers thought MMIs fair, 23% felt they needed more specific training	7.5
El Says et al. 2013 [62] Research paper	Saudi Arabia 1 Medical School	Quantitative	MMI	Applicants and interviewers N=UTD, RR = UTD	UTD	UTD	MMI was acceptable to both students & faculty	3
Eva et al. 2004 [63] Research paper	Canada 1 Medical School	Quantitative	MMI (voluntary MMI did not actually contribute to selection)	Applicants *N* = 115 RR 98% Assessors *N* = 40, RR = UTD	Questionnaire with Likert scale responses and free text comment boxes	Mean ratings and standard deviations of Likert scale responses between 1 (definitely not) to 7 (definitely).	Applicants & Assessors felt candidates were able to accurately portray themselves (5.64, 5.7) adequate advance instructions (5.87, 6.1) clear station instructions (5.84, 6.2) respectively. Free text: applicants wanted more time and interviewers wanted more training	**7**
Eva et al. 2004 [64] Research paper	Canada 1 medical School	Quantitative	MMI (voluntary MMI did not actually contribute to selection)	Applicants *N* = 54, RR UTD Interviewers *N* = 36 RR = UTD	Questionnaire - 8 items Likert scale responses and free text comment boxes	Mean ratings and standard deviations on 7 point Likert scale (1 = definitely not, 7 = definitely)	Applicants and assessors felt that candidates could accurately portray themselves (5.26, 5.23), adequate advance instructions (5.84, 5.54) clear station instructions (5.86, 5.53) respectively. Applicants found the MMI difficult (4.05).	7
Eva & Macala 2014 [65] Research paper	Canada 1 medical School	Quantitative	MMIs-(voluntary MMI – comprising – free form stations, behaviour interview (BI) & SJT type interview	Applicants *N* = 41 RR = UTD Interviewers N = 48 RR = UTD	Questionnaire - 5 items for applicants and similar version for interviewers	Mean ratings, standard deviations on 7 point Likert scale (1 = definitely not, 7 = definitely, or modified slightly to match the question)	Applicants: Free form stations more anxiety (*p* < 0.05), difficult (*p* < 0.01). No difference between interviewers’ views of the 3 station types re difficulty, clear instruct-tions, difficulty for candidate, but slightly lower rating for ability to judge applicant ability on BI(*p* < 0.05)	8
Gale et al. 2010 [66] Research paper	UK 1 Postgraduate training programme – anaesthetics	Quantitative	Selection Centre – 4 stations: Structured interview, Portfolio, Presentation, Simulation.	Applicants *N* = 178 RR 79% over two years 2007/ 2008 Assessors –*N* = 24 RR = UTD	Questionnaire -	Mean and standard deviations on 5 point Likert scale (1, poor; 3, satisfactory; 5, excellent)	All four methods were positively rated by applicants & assessors for relevance (3.6–4.7), fairness (3.9–4.4), opportunity to demonstrate ability (3.6–4.2). Both groups rated the simulation station significantly higher for relevance, opportunity to demonstrate ability (*P* < 0.001)	7
Goulston & Oates 2009 ^a^ [47] Report	Australia 1 Medical School	Mixed methods	MMIs, Academic record/ GPA Aptitude test/ GAMSAT	Extensive consultation with applicants, medical students, Faculty, alumni, Health Services, clinical training, professional bodies & more N=UTD, RR = UTD	Focus groups, submissions, email surveys, face to face interviews, invited submissions	Medical School report –collating the submissions into 29 recommendations	Stakeholders confirmed a commitment to widening diversity; approved MMIs as the interview tool; recommended including community interviewers; affirmed final selection ranking based on GPA 25%, GAMSAT 25%, MMI 50%	4
Griffin et al. 2008 [67] Research paper	Australia 1 medical school Medical School	Quantitative	MMIs, Aptitude test/ UMAT	Applicants – *N* = 287 RR =84%	Questionnaire survey	Perceptions of the usefulness of coaching, previous interviews experience and practice run on MMI and UMAT performance	Just over half (51.4%) had accessed coaching. Those who had attended coaching rated it more helpful than those who had not (*P* = 0.001). A MMI practice run was considered most effective way to prepare for MMIs compared with coaching or other interview experience.	8.5
Gula et al. 2014 [68] Abstract	Canada 1 medical school	Quantitative	Standardised interviews	Applicants N=UTD RR = UTD Interviewers N=UTD RR = UTD		Views on atmosphere of interviews, confidence in interviews	Standardised interviews positively received by applicants and interviewers	4.5
Harris & Owen 2007^a^ [69] Research paper	Australia – 1 medical school	Mixed methods	Non-cognitive characteristics MMIs	Medical students, early graduates, health academics, clinical health workers & administrators *N* = 105, RR UTD Also surveyed applicants, post MMI. N=UTD/RR = UTD	Using q method-ology stakeholders ranked non-cognitive characteristics. No details of survey	Ranking of statements Applicant feedback simply summarised – no details provided	6 factors emerged & used to develop the MMI: Love of medicine and learning, groundedness, self-confidence, balanced approach, mature social skills and realism. Applicant feedback overall positive.	7
Henry 2006 ^a^ [70] Research paper	USA 1 premedical preparatory programme	Quantitative	Aptitude Test/ MCAT Academic record/ GPA	*N* = 97 premedical students	Modified version of Perceived Educational and Career Barriers Inventory	Mean responses to 31 items –Likert-type response scale consisting of strongly disagree (1) - strongly agree (5).	Barriers- Not having a high enough GPA (22% mean 2.38) and MCAT (38% mean 2.94) were seen as the most significant barriers. Letter of recommendation was not seen as high a barrier (78% did not see it as barrier, mean 1.78).	8
Hofmeister et al. 2008^a^ [48] Research paper	Canada Residency Programme- Family Medicine at 2 Medical Schools	Embedded mixed methods	MMI	Applicants *N* = 69,RR = 97% MMI Interviewers *N* = 31, RR = 94%	Survey with quantitative and qualitative components	Analysis of Likert scale responses (5 point Likert scale 1 strongly disagree, 5 strongly agree) and qualitative data content analysis	Applicants: Preferred MMIs over other interviews (4.6), free from culture (4.6)/gender (4.8) bias. Interviewers: well prepared (4.1) fairness (3.9)/ ability to differentiate (3.6). MMIs helpful assessing professionalism. Needed more time to calibrate.	8.5
Hopson et al. 2014^a^ [71] Research paper	USA 3 Emergency medicine training sites	Quantitative	MMI	Emergency Medicine (EM) interns *N* = 71 RR =98.6%.	Pre and post experience surveys	Mean Likert responses using five point scale	MMI as part of an interview process would negatively influence their decision to accept offer of interview mean 2.7 pre and 2.8 post MMI. Preference for combined approach of mixed MMI and TI. MMI score did not correlate significantly with preference for MMI. MMI was viewed as an accurate assessment of communication skills (3.3), problem solving skills (3.3)	10
Humphrey et al. 2008^a^ [72] Research paper	UK 1 post graduate deanery Paediatric training programme	Quantitative	MMI	Applicants *N* = 72, RR 75%. Interviewers *N* = 15, RR 100%.	Questionnaires- Cronbach alpha for applicant and interviewer survey 0.88 &.62 respectively	Means, standard deviations Likert scale responses between 1 (strongly disagree) to 6 (strongly agree) and free text comments	Applicants: Fairness 4.3, organised well 5.1, understandable questions 4.8, adequate info 4.4, fairer than traditional interview 4, preferable to traditional interview 3.7, (IMGs preferred MMI significantly more) Wanted more information (*n* = 8), more time (*n* = 3). Gender, age or previous MMI experience did not impact opinion. Interviewers- MMI better than TI 4.8, fair 4.4, needed more stations 4.3, selects best candidates 4, tests appropriate range of competencies 3.6, performance at interview predicts future performance 3.2	9.5
Husbands et al. 2014 ^a^ [73] Abstract	UK Undergraduate Medical school	Quantitative	SJT	Medical School applicants *N* = 200 RR = 36.2%	UTD	Applicants perceptions of relevance and validity of SJT summarised – no details	Most applicants (no details) agreed that SJT appeared relevant and valid	3.5
Jauhar et al. 2008^a^ [74] Rsearch paper	Scotland National survey	Quantitative and open ended comments	Shortlisting / Traditional interviews (TI)	Doctors on Psychiatry training programme *N* = 123, RR =61.5%	Questionnaire - both open- and closed-ended questions, using a Likert scales	Percentage responses to Likert scale questions	76% lack of confidence in shortlisting process with no significant difference between successful / unsuccessful candidates. 45% thought interviews were invalid. Poor communication & inadequate feedback were problematic. 92% felt references should be available at interviews 63% favour structured references.	8.5
Jayasuriya et al. 2012 ^a^ [37] Abstract	UK 1 medical school	Qualitative	Not specified	Medical Students (N=UTD)	Focus groups (N=UTD)	Students perceptions	Students were aware of the components of selection but unsure how they were used. Inconsistency in student advice. Preferred non-academic interviews that used personal statements and communication scenarios	NR
Johnson & Elam 2001^a^ [75] Short research report	USA 1 Medical School	Quantitative	Letters of recommendation (LOR)	Admission committee members *N* = 14, RR 93% Premedical advisors *N* = 42 RR = 87.5%.	Using example letters of recommendation, rated usefulness on 5 point Likert scales from “Not at all” to “Extremely”.	Perceptions of usefulness	There was no difference between the two groups in their perceptions of usefulness and global impression of the sample letters. Both thought most helpful when they factual, descriptive and cited examples of specific behaviours.	8
Kaffenberger et al. 2014^a^ [76] Letter – original research	USA National Survey	Quantitative	Letters of recommendation (LOR)	Professors of Dermatology - *N* = 129 RR = 37%	Survey- no details	Percentage responses displayed graphically	LOR from Dermatology Professors and “Physicians I know” considered more reliable than other sources. Perceived problems with LOR are frequently having difficulty in ascertaining the strength of recommendation and reluctance to give honest account of weaknesses	6.5
Kelly et al. 2014 ^a^ [77] Research paper	Ireland 1 Undergraduate medical School	Quantitative	MMI (experimental, did not contribute to selection) Aptitude Test / HPAT-Ireland	First Year Medical Students *N* = 71 RR = 65% MMI Interviewer *N* = 24, RR = 49%	Electronic questionnaire survey	Percentage responses	90% students agreed that the MMI content was relevant, 60% felt content of TIs or HPAT-Ireland (38%) were relevant, 73% felt MMI suitable for selection, 79% supported academic record. 75% of interviewers felt that MMI was relevant, reasonably tested candidates’ ability (79%). The majority (71%) thought MMI would be a useful addition to selection	8
Kelly et al. 2014 b^a^ [38] Research paper	Ireland 1 Undergraduate medical School	Qualitative	Aptitude test/ HPAT-Ireland	Qualified doctors from various disciplines (n = 15)	Interviews – analysed using principles of grounded theory	Perceptions of job relevance, acceptability of HPAT-Ireland	Sections 1 and 2 perceived to have good job relatedness, but Section 3 non-verbal reasoning, criticised. Split views on acceptability, with those opposed being principally concerned re possible negative impact on diversity.	NR
Kleshinski et al. 2008^a^ [78] Research paper	USA 1 medical school	Quantitative	Interviews - regarding the value of including professionalism/ethics scenarios in selection interviews	Faculty interviewers *N* = 91 Applicants *N* = 107, RR = 54%	Survey	Percentage responses to questionnaire items with five point likert scale responses to statements	Applicants: 74% asking about professionalism positively impacted & 76% agreed it enhanced their view of the medical school. 88% agreed it was important to include in selection interviews. Applicants more positive than interviewers re importance of including professionalism (88% versus 69% *p* = 0.0001).	7.5
Koczwara et al. 2012 [79] Research paper	UK Post graduate GP training in one geographical area	Quantitative	Cognitive ability tests clinical problem-solving test (CPST), situational judgement test (SJT)	Applicants *N* = 249, RR 96%	Validated candidate evaluation questionnaire	Percentage and frequency of responses	Cognitive ability tests: 30% not fair-fair, 35% content not appropriate, 54% not relevant. By contrast figures from the overall 2009 GP applicant pool (*n* = 2947) showed that the CPST and SJT were regarded as relevant by 89, 63%, appropriate 85, 68% and fair 85 and 53% respectively.	8
Kumar et al. 2009 ^a^ [39] Research paper	Australia & Canada 2 Graduate Entry Medical Schools	Qualitative	MMI	MMI Interviewers *N* = 37, took part in focus groups MMI Interviewers (*n* = 75, RR 48%)- completed a survey Applicants (*n* = 442; RR = 91%) completed a survey	6 Focus groups and open-ended survey	Framework analysis	Very positively viewed. Candidates valued interviewer independence & multiple opportunities,, but felt time pressured and absence of opportunity to present their motivations. Interviewers less anxious about decision making, but concerns re measuring communication skills and lack of opportunity to bench their marking.	NR
Kumwenda et al. 2013^a^ [80] Research paper	UK 6 Medical schools and 1 dental school	Quantitative	Application including personal statement / UCAS	First year entrants to medical and dental school *N* = 432 RR = 34%	Online questionnaire	Average and percentage responses Cronbach alpha 0.77.	66% suspect peers stretch the truth, 16% deceptive practice is common, 84% lying unacceptable, 949% exaggerating on UCAS is dishonest but 14% think part of the admission game (males agree more *p* < 0.05)	9
Lambe et al. 2012 [81] Research paper	UK 1Medical School	Quantitative	Aptitude Test/ UKCAT	Applicants N = 787, RR = 66%,	Online questionnaire	Percentage responses	86% thought that you can prepare for the UKCAT, 44% felt that advice on the UKCAT was confusing, 55% felt test was fair and 44% agreed it was relevant	6.5
Lievens 2013 [82] Research paper	Belgium National survey Medical and dental undergraduate	Quantitative- longitudinal multiple cohort study (1999–2002)	SJT & Cognitive tests	Applicants N = UTD, RR 61.8%	Validated questionnaire	Mean, Standard deviation of responses on a 5-point Likert scale (1 = strongly disagree, 5 = strongly agree).	Mean rating for the face validity of the SJT (3.19 SD 0.88) was significantly higher than cognitive test (2.76 SD 0.68) *p* < 0.01. SJT viewed as significantly less difficult than cognitive tests.	8.5
Lievens & Sackett 2006 [83] Research paper	Belgium National survey Medical and dental undergraduate selection	Quantitative	Two formats of SJT video based versus written formats & Cognitive tests	Applicants from two cohorts (*N* = 638, RR 55%; *N* = 1078, RR 61%)	Validated questionnaire Cronbach alpha (0.66, 0.76)	Mean, Standard deviation of responses rated on a 5-point Likert scale ranging from 1 (strongly disagree) to 5 (strongly agree).	No significant difference between the mean face validity perceptions of the video SJT (3.41) and written SJT (3.44). Both significantly higher than face validity of the cognitive test (2000 = 2.75, 2003 = 2.79).	10.5
Lubarsky &Young 2013 [84] Abstract	Canada 1 hospital Neurology residency program	Quantitative	MMI	Applicants N = 29 RR = 94% MMI interviewers no details provided	UTD	UTD	Both applicants and interviewers felt MMIS allowed applicants to showcase their unique attributes and skills, but that the process felt somewhat ‘impersonal’	4
Marrin et al. 2004^a^ [49] Research paper	Canada 1 Medical School	Quantitative	Key qualities of medical selection process, no particular tool identified	Admission stakeholders- *N* = 277 comprising Faculty, students and community Mean RR across the stakeholder groups 71%	Paired comparison approach-	Z scores for probability of each characteristic being chosen from the pairing	No significant difference between stakeholders. Fairness (mean z score 0.92), Validity (mean z score 0.87), comprehensiveness (0.44), accessibility (0.1), defensible (− 0.3), leads to diversity (− 0.31), affordable (− 0.8), public statement (− 0.9).	8.5
Mathers & Parry 2010^a^ [40] Research paper	UK 3 Medical Schools	Qualitative	Not specified	Older mature students (N = 15)	Unstructured one to one interviews	Framework analysis	Decision to apply made after careful consideration of university location/ access to family support/ identity and fit were key. Inflexibility and uncertainty of process/ UCAS inflexible/ Risks involved in making the application. Financial cost	NR
Milne et al. 2001^a^ [85] Research short report	USA 1 Medical Residency Programme	Quantitative	Interviews	Medical Interns n = 53, RR = 87%.	Questionnaire survey	Percentage responses to categories of five point Likert scale responses from 1 (strongly disagree) to 5 (strongly agree).	Interviews viewed as a chance to learn more about programme (84%), sell myself (80%), determine faculty satisfaction with institution (76%) and their own interest in the prog (71). 86% felt interview was necessary, 93% believing no interview was unacceptable.	8
Mitchison 2009^a^ [86] Research paper	UK 1 Post graduate deanery	Quantitative	Selection centre with 3 types of station: structured interview/ case based discussion/ simulated patient	Assessors-*N* = 53, RR 77%.	Feedback questionnaire	Frequency & percen-tage responses. Note – feedback given per station type, with some assessors responding to more than 1 station	19/21 assessors found the Structured interview useful, 21/24 the communication station, 27/27 case based discussion. 69% felt SC was an improvement on TI, 4% felt it was worse. Positive free text: fairness &objectivity. Negative: inflexibility to explore other issues	7
Monroe et al. 2013^a^ [87] Research paper	USA / Canada Large scale survey of 142 medical school	Quantitative	Aptitude test / MCAT Academic record / GPA Interviews Letter of recommendation	Admission Deans from all US and Canadian medical schools using MCAT *N* = 120 RR = 85%	Online survey − 69 items - derived from qualitative interviews in 8 medical schools	Means Standard Deviations, frequency of responses. 5 point Likert scale (1 not important – 5 extremely important)	Rating differed depending on the stage of the process- MCAT & GPA viewed most important for shortlisting to interview but less important in the decision of who to admit where interview (mean rating 4.5) & letter of recommendation (3.7) more valued (no *p* value given), followed by GPA (3.6), community service/volunteering (3.5), MCAT (3.4) and personal statement (3.3)	10
Niyomdecha et al. 2012^a^ [88] Abstract	Thailand 1 medical school	Quantitative	MMIs	Medical students and instructors N=UTD, RR = UTD	UTD	UTD	88% of instructors thought MMI process was good and 100% of students thought MMI was fair	4
O’Brien et al. 2011 [89] Research paper	UK 1 Undergraduate medical School	Quantitative	MMI (experimental, not used for selection) Standardised interviews (SI)	Applicants *N* = 47,RR = UTD Interviewers-N=UTD RR = UTD	Questionnaire survey with free text comment boxes	Means, standard deviation, of responses on a 5 point Likert scale scoring	No statistical difference between Interviewers’ ranking of SI and MMI with respect to overall opinion, fairness, accuracy and ability to pick best candidate. School leavers: MMI more accurate, less difficult than SI (*p* = 0.03 and 0.01). Graduate entrants: MMI more difficult than SI (*p* = 0.005).	7
O’Flynn et al. 2013 ^a^ [90] Research paper	Ireland National survey	Quantitative	Academic Record – School leaving certificate examination Aptitude test - HPAT –Ireland	Guidance counsellors *N* = 187, RR = 15%.	Questionnaire – 26 items and free text comment s	Percentage responses to likert scale answers and simple content analysis of free text comments	52% in favour of the introduction of HPAT-Ireland, 49% felt new system was fair. Those opposed were concerned re negative impact on socially disadvantaged. Majority felt non-verbal reasoning least relevant.	7
Patel et al. 2011^a^ [91] Abstract	USA Post graduate − 1 medical residency programme	Quantitative	Group Interview	Applicants *N* = 77 RR = 38%	Online anonymous questionnaire – 15 items	Percentage responses to survey questions	75% liked group interviews, 62% would recommend continued usage. 89% felt group interviews effective. IMGs felt they had much harder time impressing interviewers than local candidates (*p* = 0.004)	6
Patterson et al. 2009^a^ [92] Research paper	USA 1 medical school with mission to recruit from and serve underserved populations	Quantitative	Aptitude test/MCAT Academic record / GPA	Applicants - American Indians and Alaskan natives *N* = 34, RR = 38%, from three chorts. Included and compared accepted (*N* = 21) and rejected (*N* = 13) applicants .	Questionnaire developed from research and pilot – containing numerical and open ended questions	Frequency and percentage responses, chi square and t-test, and groupings of free text comments	Access to supports was limited for both groups but rejected applicants had significantly less support (*p* < 0.05).MCAT viewed as a barrier by 65% & financing the application by 42% (rejected more so, *p* < 0.05) Free text comments identified 3 obstacles – finance including cost of MCAT preparation, lack of time to volunteer/ build a CV etc. as working & discouraging/wrong information	8
Patterson et al. 2011 ^a^ [7] Research paper	UK Postgraduate training in General Practice - national survey	Quantitative	SJT Clinical problem solving test (CPST) Selection Centre (SC) comprising: Simulated patient Group exercise Written exercise	Applicants to GP training- 3 cohorts 2007–09. *N* = 9067, RR 56%	Online and paper questionnaire survey.	Mean, Mode Standard deviation of responses Cronbach alpha survey 0.7–.94.	Shortlisting: SJT viewed very job relevant but CPST as more so *p* < 0.001. Perceptions of fairness (formal test characteristic and interpersonal treatment) were good. Selection centre- All three tasks positively viewed in terms of job relevance, SP more positively *p* < 0.001. Perceptions of fairness high	9.5
Patterson et al. 2013 [93] Abstract	UK Large scale survey	Quantitative	SJT	Candidates to Foundation Year Training Programme N=UTD, RR = UTD	UTD	Candidate reaction	Feedback from candidates indicate SJT relevant and fair	3
Randall et al. 2006 [94] Research paper	UK 1postgraduate Paediatric Deanery	Quantitative	Selection centre (SC) comprising group discussion, simulated patient written exercise	Applicants *N* = 27, RR UTD	Questionnaire survey	Frequency of responses on 5 point Likert scale (1 strongly disagree to 5 strongly agree)	*N* = 24agreed that the SC content was appropriate, 24 agreed more relevant than other selection tools & provided better opportunity to demonstrate their skills	6
Razack et al. 2009^a^ [50] Research paper	Canada 1 Undergraduate medical school	Embedded mixed methods	MMI	Applicants -International and non-local. *N* = 82 RR = 82%. Unsuccessful applicants *N* = 50 RR = 60%. Interviewers *N* = 38 RR = 100%	Questionnaires with quantitative and qualitative components	Mean ratings and standard deviations of Likert scale responses between 1 (strongly disagree) to 6 (strongly agree). Content Analysis of free comments.	Applicants: MMI more fair than SI (*p* = 0.001) & more effective at evaluating non-academic aptitudes (*p* = 0.001) more stressful (*p* = 0.016). Interviewers’ mean scores: Fair 5.2, effective 5.1, appropriate for use with home and international applicants 4.9, transparent 5.2. Concern candidate difference may affect performance, misses some of benefits of TI, some practical issues.	8
Rich 2011 ^a^ [95] Abstract	UK 1 medical school- with a widening access agenda	Quantitative	Traditional Interviews (TI)	Medical student admitted via a widening access (WA) route N = UTD RR = UTD Interviewers n = UTD, RR = UTD	Questionnaire	Satisfaction with TI for widening access in selection	49% students in early clinical years felt TI should be retained. Only 25% of students in clinical years and 20% of interviewers agreed favouring multi station interviewing.	5
Rodgerson et al. 2013 ^a^ [96] Abstract	UK Medical / Dental School	Quantitative with free text	MMI Traditional Interview	Medical *N* = 451) and dental applicants (*N* = 224) RR = 75%	Online survey post	Percentage responses and mean ratings of responses using five point Likert scale	94% agreed MMI suitable for assessing potential 45% agreed TI suitable. MMI more favourable re enjoyment, stressfulness & fairness. 80% gave free text comments relating to fairness, with 40% approving of the opportunity to impress multiple interviewers.	4.5
Samarasekera et al. 2014 [97] Abstract	Singapore 1 medical school	Quantitative	SJT and Focused skills assessment	Applicants and assessors N=UTD, RR = UTD	UTD	UTD	92% of candidates happy with format. 82% assessors positive perceptions of the process which Evaluated empathy, communication, integrity, general knowledge, resilience, personality profile	5
Stagg & Rosenthal 2012^a^ [41] Research paper	Australia 1 Medical School	Qualitative	Not specified	Community Members and Members of the rural based Community Liaison *N* = 12	Semi structured individual interviews	Thematic analysis	Overwhelmingly saw involvement in selection of students as positive. Opportunity for professional &personal growth; responsibility to represent the broader community; protecting the student and public interest and self-interest in shaping the future workforce.	NR
Stevens et al. 2013^a^ [51] Research paper	Ireland 3 medical schools	Mixed methods – embedded	Aptitude Test/ HPAT –Ireland	First Year medical students *N* = 291, RR = 77%,	Questionnaire survey	Percentage responses and simple content analysis of free text	Almost all support academic record as suitable tool, 78% interviews, 74% personality tests, 68% adjunct admission tests. International students more likely to support interviews, knowledge about course, references and personal statements (all *p* < 0.01), Of those who had sat HPAT (*N* = 175) – 76% felt it fair, 37% felt it was easier for males, 32% felt non-verbal reasoning section irrelevant . 54% had accessed prep course, of these 79% felt it improved performance	8
Tiller et al. 2013 [98] Research paper	Australia Graduate entry medical & dental school	Quantitative	Internet based MMI (iMMI)	Applicants *N* = 119 RR = 41% Interviewers *N* = 78, RR = UTD	Online survey	Percentage responses and mean ratings to responses using five point Likert scale(5 = very satisfied, 1-very unsatisfied)	Mean satisfaction ratings with use of skype technology 4.25, overall interviews process 4.2, being interviewed online as part of overall selection process 4.10, video quality 4.09, audio quality 4.08. 68% would prefer an in-person MMI and 32% a skype interviews. 78% of Interviewers satisfied with the iMMI, 71% with the technology. Free text comments re concerns re operational & technical issues	8
Turner & Nicholson 2011^a^ [42] Research paper	UK 1 Undergraduate Medical School	Qualitative	Written application / UCAS Personal Statement/ Letter of reference	Medical school selectors –in three focus groups- clinical / non-clinical and lay members N = 17	Focus groups and document review	Thematic framework analysis and triangulation with recorded reasons	Four themes: Work experience/ commitment to study medicine/ teacher reference/ personal statement. Most common reason for rejection was poor medically related work experience. Teacher reference viewed as influential esp. for rejection but hard to interpret. Personal statement – useful but considered highly subjective. Ideal candidate extremely difficult to judge	NR
Uijtdehaage et al. 2011 [99] Research paper	USA 1 medical School Specialised to develop leadership & serve disadvantage	Quantitative	MMI	Applicants Cohort 1: *N* = 76, RR =100% Cohort 2: *N* = 77, RR =99% Interviewers *N* = 26 RR = 93%	Questionnaire surveys – 8-10 items	Mean ratings and standard deviations of Likert scale responses between 1 (definitely not) to 7 (definitely). Cohort results presented separately	Applicants: able to present abilities (5.6, 5.7), adequate instructions (6.2, 6.5), sufficient time (3.7, 4), free from gender (6.6–6.7) or cultural bias (6.3, 6.6), stressful (4.2, 3.7) . Interviewers – accurate portrayal (5.6), prepared (6), clear instructions (6.2), adequate time (4.7), allow differentiation (5.7), Overall fair (6.2)	9
UKCAT Consortium 2009/2010 [100] Report	National Survey UK Medical Schools using UKCAT	Quantitative	UKCAT / Aptitude	Applicants *N* = 6821, RR =27%	Questionnaire	Percentage responses and majority opinions	Considered a difficult test. Unconvinced it tests right attributes. 40% felt their college or school were not well informed about UKCAT/ ¾ had used online practice materials/ books, and found them useful. 90% happy with testing environment.	6
UKCAT Consortium 2011 [101] Report	National Survey UK Medical Schools using UKCAT	Quantitative	UKCAT / Aptitude	Applicants RR = 19.5%	Questionnaire	Percentage responses and majority opinions	44% found out it from websites /prospectuses. 33% found out from their schools. 36% of candidates from independent schools rated their advice as good or very good, only 18% from comprehensive schools agreed with this. Majority very supportive of practice tests (93%) and books (90%). Timing in the test is crucial.	6
Vermeulen et al. 2012 [102] Abstract	Post Graduate GP training. Nether-lands	Quantitative	Behaviour specific interviews, knowledge test, SJT and simulated consultation versus traditional interviews (T)	Applicants *N* = 47 RR = UTD	UTD	UTD	Both TI & behaviour specific interviews were considered job relevant & fair/ but latter offered better opportunity to show competencies. Both SJT and knowledge based test were considered job relevant. SJT considered fair (95.7%), simulated consultation (78%) knowledge based test (64%).	4.5
Waheed et al. 2011^a^ [103] Research paper	Pakistan 1 medical school	Quantitative	Interviews that include a scenario based on professionalism as part of the interview	Medical Students *N* = 100 Faculty members *N* = 100	Students and faculty attended a lecture about profesion-alism scenarios that could be included in interviews and then discussed these and completed a survey	Frequency of responses	77% of students highly positively influenced by the lecture compared to 10% faculty, 85% students / 76% Faculty agreed it influenced their impression of Medical school values Faculty more likely to feel important to include such scenarios in admission interviews (*p* = 0.01)	7
Westwood et al. 2007^a^ [104] Research paper	UK 1 Post graduate Deanery Cardiology	Quantitative	Structured Interviews	Applicants *N* = 94, RR = 80%.	Questionnaire	Median and interquartile range of Likert scale responses between 1 (strongly agree) to 5 (strongly disagree)	Satisfaction rating high, (2), objective (2), appropriate duration (2) offered sufficient scope to express individuality (2) and was relevant to the job (2).	7
White et al. 2011^a^ [43] Research paper	Canada 1 Medical School	Qualitative	Essay	Applicants - *N* = 20	Review of 240 randomly selected essays and interviews. Qualit-ative analysis using modified grounded theory	How applicants approach writing the essay	Applicants expressed the idea that they had approached the essays as a way to “show themselves” and “tell their own story” in a subjective way which they felt was missing from other parts of the admission process.	NR
Wilkinson & Wilkinson 2013 [105] Research paper	New Zealand 1 Medical School	Quantitative	Aptitude Test/ UMAT	Medical Students – two cohorts 2010/2011 *N* = 263 RR = UTD	Online survey of self -reported- forms of preparation used for UMAT and received confidence	Percentage response rates, comparisons of scores mean and standard deviation	Commonest forms of preparation were ACER practice materials, MED Entry course, and student led tutorials. Students who took a MED Entry course had significantly higher confidence (mean diff Likert score 0.6), No significate differences for those taking student led tutorials. Moderately strong positive correlation between amount of money spent and confidence *r* = 0.3, *p* < 0.001.	10.5
Wright 2012 [44] PhD Thesis	UK 1 Medical School	Mixed Methods thesis, with Qualitative strand used to explore stakeholder views	UK Medical School Admission processes- personal statements, interviews	Medical Students (n = 13)	Interviews - Qualitative analysis using Framework analysis	Students’ views of influences on decision to apply to medical school and preparedness	Family & School were highly influential on decision, support for application activities such as work experience, preparing personal statements and interviews practice. Students from medical /professional backgrounds and fee paying schools were better supported/prepared.	NR
Ziv et al. 2008 [106] Research paper	Israel 1 Medical School	Quantitative	Selection centre/ known as MOR	Applicants (two cohorts) *N* = 510 RR = 90.6% - MOR Raters – largely Senior Faculty members *n* = 352 RR = UTD	Questionnaire	Frequency of responses with four point Likert scale	76% of applicants rated MOR as fair 76% felt they had opportunity to express their capabilities 85% (*n* = 299) of rater found it fair. 92% rated MOR assessment items as appropriate	7

Key: NR - not relevant because qualitative study, UTD –unable to determine as insufficient information provided

^a^ Studies where establishing stakeholder view was principal aim

### Risk of Bias

The included studies ranged from qualitative to quantitative and mixed methods and were predominately descriptive study designs. Therefore, performing a risk of bias-assessment across studies was not possible, and we focused instead on assessing the quality of the reporting of data and outcomes of the studies using the MERSQI tool.

### Study designs

A data display matrix summarising the main research findings, and MERSQI scores of the studies included in this review is presented, in alphabetical order (see Table 1).

Included records comprised eight qualitative studies [37–44]. Seven were mixed methods studies [45–51]. The remaining records were quantitative [52–106].

Twelve records were abstracts [37, 54, 68, 73, 84, 88, 91, 93, 95–97, 102]. Two were peer-reviewed short research reports [75, 85], one PhD [44], one commissioned report [47], one letter describing original research [76]. The remaining were peer-reviewed original research papers.

Twenty-two records were from studies conducted in the UK, 12 in Canada, 12 in USA, 6 in Australia, 5 in Ireland, 2 in New Zealand, 2 in Belgium, 1 in Australia/ Canada, I in USA/Canada, and 1 each in Israel, Pakistan, Netherlands, Singapore, Thailand, and Saudi Arabia respectively.

The sample size ranged from a minimum of 14 to a maximum of 9067 (mean 397, median 91) excluding qualitative studies. Twenty-nine records included the views of more than one stakeholder group, most commonly applicants and assessors.

### Synthesis of results

The research largely explored the views of three main stakeholder groups: a) applicants; b) selectors and c) medical students.

#### The views of applicants

Applicants constituted the most researched stakeholder group (45 records).

#### Interviews including multiple mini interviews (MMIs)

Applicants’ views of MMIs, both at medical school and residency training levels, have been extensively surveyed internationally, most likely reflecting their novelty, within the timeframe of this review. The research was generally of good quality (10 records with a MERSQI score over 8), achieving high response rates (9 records with response rates over 75%) and a reasonable sample size (9 records where n = ranged 69–324).

Applicants are on the whole supportive of MMIs. They perceive that they are generally fair, relatively free of gender or cultural bias, provide adequate opportunity to present their abilities and strengths and that the quality of advance information and clarity of instructions are good [45, 48, 50, 55, 61, 63, 72, 99]. Applicants indicate a preference for MMIs over traditional interviews [45, 48, 60, 72, 89, 96]. One paper included the views of a small number of unsuccessful applicants, and found that the majority still commented positively on MMIs [50]. Applicants value the perceived independence of interviewers and the authenticity of MMIs [39, 45, 96]. In particular the multiple opportunities for applicants to demonstrate abilities appears influential on positive reactions [39, 55, 61, 63, 99]. The chance provided by MMIs to “*redeem*” oneself has been positively noted [39].

Applicants’ reported some misgivings with respect to MMIs. Some applicants found MMIs more difficult [89], and more stressful [61], than standardised interviews, while others were concerned that MMIs favour highly communicative applicants [39]. When compared to ratings of other aspects of the MMIs, applicant satisfaction with allotted time was slightly lower [39, 55, 60, 63, 99].

Applicants’ views of other interview techniques were also positive; with one small study reporting that the majority of participants (93%, *n* = 53) believed that any selection process which did not include interviews would be unacceptable [85]. Standardised interviews have been positively received by applicants in one Canadian medical school [68]. Technological advances have made web based interviewing a possibility and two studies report positive applicant reactions to this approach [57, 98]. Applicants also perceive interviews as an opportunity to glean valuable information about the values and ethos of the school or programme to which they are applying [56, 78, 85]. One study reported favourable levels of applicant satisfaction with group interviews, however international applicants felt they would struggle to impress interviewers by comparison with local candidates (*n* = 77, response rate 37.8, *p* = 0.004) [91]. Only one paper was identified that reported negative applicant reaction to panel interviews, and in this paper criticisms related mostly to inadequate levels of post interviews feedback [74].

#### Situational judgement tests

A small number of studies (*n* = 6) have explored applicant perceptions of Situational Judgment Tests (SJTs). In terms of quality the MERSQI ratings range from 3 to 10.5, with 4 records with a MERSQI score between 8 and 10.5; response rates were provided by five records and ranged from 36 to 96% and sample sizes, where indicated ranged from 200 to 9067. Two national studies in Belgium found that medical school applicants rated SJTs as having significantly better face validity than aptitude tests [82, 83]. Studies of medical school applicants, foundation year doctors and two studies of applicants to UK general practice training confirmed these positive applicant reactions to the relevance and job relatedness of SJTs [7, 73, 79, 93].

#### Selection centres

Likewise a small number of studies reported positive reactions to Selection Centres (SCs). Applicants consistently consider SCs to be fair, appropriate and to offer adequate opportunity to demonstrate skills and abilities [66, 94, 97, 102]. SCs rate very positively in terms of of job relevance overall with simulated patient stations being viewed most positively [7, 79]. SCs are not often used for medical student selection, however two examples were located (Singapore and Israel) and both studies report high levels of applicant acceptability [97, 106].

#### Aptitude tests

Applicants’ acceptance of aptitude tests was somewhat less positive. Under-represented and minority applicants view the Medical College Admissions Test (MCAT) as a barrier to their chances of admission [70, 92]. Medical school applicants considered the UK Clinical Aptitude Test (UKCAT) difficult and were generally unconvinced of its relevance [81, 100, 101]. Conversly, in one study over half of respondents (55%, *n* = 787) thought that the test was fair [81].

#### Other selection methods

Only one paper was identified that explored medical school applicants’ views of the biographical essay [43]. Applicants described approaching the essays as a way to “show themselves” and “tell their own story” in a subjective way which they felt was missing from other parts of the admission process.

No article in the timeframe 2000–2014 specifically evaluated applicants’ approval of the use of academic record perhaps reflecting the long-established practice and evidence supporting their use. Likewise we did not identify any records of applicants’ views of personality assessment or references.

In summary, applicants’ views of specific selection methods have been widely surveyed, with the preponderance of evidence relating to applicants’ opinions of newly introduced tools. Applicants appear to be consistently supportive of interviews and MMIs in particular. There is reasonable emerging evidence that both SJTs and SCs are also well regarded. Conversely aptitude tests were not as well supported by applicants. There were significant gaps with respect to applicant views of other selection tools.

#### The views of selectors: interviewers, faculty and admissions committee members

Thirty seven records included the views of selectors (mean and median MERSQI scores of these records =7). Selectors comprised interviewers, faculty and admission committees constituting persons from a wide variety of backgrounds, both clinical and non-clinical, who share a responsibility for particular aspects of medical selection. These individuals serve variously to appraise written applications, letters of reference, personal statements; serve on interview panels or assess MMI stations; or develop and assess performance on SJTs and SCs. In this fashion they contribute to either shortlisting applicants or a final selection decision. Fairness, validity and comprehensiveness are viewed as crucial aspects of the selection process [49]. A strong sense of social accountability motivates community members and lay persons to become involved in the selection process [41].

#### Interviews including MMIs

Interviews are considered a stalwart by selectors to medical schools [87]. Similarly, in a large study examining stakeholders’ views of selection methods to Australian medical schools, interviews were viewed as the most valid selection method overall [54].

A large number of studies have evaluated selectors’ opinions with respect to MMIs. Interviewers ranked MMIs highly in terms of perceptions of fairness [45, 48, 50, 61, 72, 99]. Importantly, interviewers felt that MMIs allowed them to accurately evaluate applicants and that the scoring mechanisms allowed them to adequately differentiate between candidates [45, 48, 55, 60, 63, 77, 99]. The multiple assessment opportunities afforded to candidates and the multidimensional assessor view meant that interviewers felt much less anxious about their own decision making [39]. Razack et al. report that interviewers found MMIs appropriate for use with home and international applicants [50]. There is some evidence that interviewers may favour MMIs over traditional interview [45, 72].

Interviewers’ concerns regarding MMIs include: a fear that it might be primarily measuring communication skills [39]; that issues including applicants’ culture, personality or language may negatively impact on performance [38, 50]; the lack of opportunity for interviewers to benchmark the scores they assign against their peers [39]; insufficient time for calibration [48], the requirement for additional training [61] and that MMIs can be a somewhat impersonal process [84].

#### Situational judgement tests

This review did not identify any records reporting selectors’ views of SJTs.

#### Selection centres

Emerging evidence suggests that selectors, in both in medical school and postgraduate residency settings, are supportive of SCs. Overall assessors rate SCs highly for relevance, fairness and opportunity for candidates to demonstrate their ability appropriateness to selection [66, 86, 106]. When compared to stations comprising structured interviews, portfolio review and a presentation station, simulated stations were rated significantly higher (*p* < 0.001) with respect to relevance to selection, opportunity to demonstrate ability and appropriateness to selection [66]. Negative findings were few but included complaints about the inflexibility of the structured approach.

### Academic records and aptitude tests

The Medical Colleges Admission Test (MCAT) and undergraduate grade point average are widely considered by Admissions Deans in North America as the two most important selection methods in the decision of who to call to interview for a medical school place [87]. However, at the decision of offers of places, interview and letters of recommendation were more influential. Cognitive ability tests and academic record were viewed by selectors, in one study, as a significant barrier to under-represented and minority applicants [53].

#### Other selection methods

Letters of reference are viewed as helpful when they were factual, descriptive and cited examples of specific behaviours [75]. In the case of postgraduate selection, they were considered more valuable when they were written by a clinician known to the selector [76]. Perceived shortcomings of letters of reference include difficulty in ascertaining the true strength of recommendation, leading to guess work and reading “between the lines” not least because of a reluctance on the part of the writer to give an honest account of candidates’ weaknesses [42, 76].

One study found that personal statements and a description of work experience were also deemed useful by selectors in terms of revealing an applicant’s depth of understanding of a medical career but considered highly subjective [42]. No records were found describing selectors’ views of personality measures.

In summary, there is reasonable evidence that selectors endorse the use of interviews in general and in particular MMIs, judging this latter tool to be fair, relevant and appropriate for selection, with emerging evidence for similarly positive reactions to SCs. Aptitude tests and academic record were viewed as most useful in the decision of who to call to interview, however they are sometimes viewed as lacking validity and acting as barriers to certain groups of applicants. The usefulness of letters of reference seems mostly to be for ruling applicants out rather than in.

#### The views of medical students

Twelve studies were identified that explored the views of medical students, distinct from those where students were directly involved in the selection process as in the group above.

#### Interviews including multiple mini interviews

Two records suggested that students prefer interviews to cognitive testing [51, 77]. International students are even more likely to support interviews (*p* < 0.01) [51]. Students appreciate the same aspects of MMIs as applicants do, describing it as relevant and suitable for use in selection [77]. One small study examined the views of students admitted through a widening access route, on the role interviews for selection [95]. Interestingly students in their early clinical years supported traditional interviews while students in the senior years felt that MMIs were more appropriate. Elsewhere mature students highlighted the importance of interviews to their sense of identity and fit with prospective medical schools [40].

#### Aptitude tests

Medical students have mixed to poor reactions to aptitude tests for selection. A good quality mixed methods study (MERSQI rating 10.8) of first year medical students in five Scottish medical schools revealed that overall, the UK Clinical Aptitude Test (UKCAT) was poorly viewed [46]. Focus group interviews showed that students felt it lacked face validity, had poor predictive validity, was coachable, potentially discriminating against less affluent applicants and that there was lack of certainty about how the test was applied by medical schools. Similarly, in a survey of two medical schools in New Zealand (*n* = 1325, response rate 65%) the majority of students were unconvinced of the importance of Undergraduate Medical and Health Professions Admission Test (UMAT), with over two thirds believing it was not fair [59]. This contrasts with findings evaluating a similar selection tool, the Health Professions Admission Test (HPAT)-Ireland which had a much more positive student reaction; in one study 76% of medical students thought it was fair and 70% felt the questions were well designed and relevant [51]. But elsewhere when compared to MMIs only 38% found HPAT-Ireland relevant [77]. One of the objections medical students have to cognitive aptitude tests is their perceived susceptibility to coaching. Stevens et al. reported the vast majority (79%) of those who had accessed commercial coaching (for HPAT-Ireland), felt it improved their performance [51]. Elsewhere students who had undertaken a commercial course in preparation for UMAT reported higher confidence levels and expected to do well, despite the evidence that coaching does not lead to significant differences in overall performance [105].

#### Other selection methods

Kumwenda et al. report that two thirds of medical students suspect peers “stretch the truth” in their personal statement as part of their written application to medical school and over 13% believe that, although dishonest, this is a necessary part of the medical school admission “game” [80]. Being from a medical family was seen as a significant advantage in gaining access to relevant work experience for inclusion in the personal statement [44]. Mature medical students indicated that they perceive the written application form to be inflexible and that there was a lack of transparency about what would constitute a good mature application [40]. No records were found exploring medical students’ views of personality assessments.

In summary, medical students appear to prefer interviews based selection methods to cognitive aptitude tests; highlighting the perceived relevance as an important influencing factor. By contrast they view the latter as being less relevant, prone to bias and susceptible to coaching. They are also unconvinced about the transparency of written applications where they believe exaggeration is both common practice and a necessary part of the selection game, with mature students perceiving them as inflexible.

#### The views of other stakeholders

We identified very few studies which sought to explore the views of other stakeholders i.e. those who are not applicants, selectors or medical students. Four such studies were identified.

One Australian study which included applicants, medical students, patients and doctors (*n* = 938) evaluated the face validity of tools used for selection to Australian medical schools and noted that medical professionals had lower confidence in the tools used than others surveyed [54]. Aptitude tests were viewed as the least valid selection method.

Three related studies were conducted following the introduction of substantial changes to national selection to medical school in Ireland, which included the introduction of an aptitude test. In a national survey of career guidance counsellors over half of supported the introduction of HPAT-Ireland [90]. Elsewhere, Dennehy et al. surveyed Irish General Practitioners who were not directly involved in selection and report that the majority (97%) strongly support academic record as a selection tool while 70% supported the use of aptitude tests [58]. Kelly et al. qualitatively explored the views of doctors, from a variety of clinical backgrounds to the same test [38]. On the whole they considered the test to have a moderately good degree of job-relatedness. However a non-verbal reasoning section was criticised by all participants, for lacking clinical relevance.

## Discussion

This review and synthesis of the evidence identifies a growing body of research into the views of stakeholders. It identified that the research largely explores the views of three main stakeholder groups: a) applicants; b) selectors and c) medical students. The emerging evidence demonstrates that there appears to be a reasonably high level of concordance of views between these stakeholder groups. Applicants support interviews, and multiple mini interviews (MMIs). There is emerging evidence that situational judgement tests (SJTs) and selection centres (SCs) are also well regarded by applicants, but aptitude tests less so. Selectors endorse the use of interviews in general and in particular MMIs judging them to be fair, relevant and appropriate, with emerging evidence of similarly positive reactions to SCs. Aptitude tests and academic records were valued in decisions of whom to call to interview. Medical students prefer interviews based selection to cognitive aptitude tests. They are unconvinced about the transparency and veracity of written applications.

The findings of this review resonate with the constructs of organisational justice theories- in particular with both procedural and distributive justice. On the whole stakeholders are supportive of interviews (in particular MMIs), SCs and SJTs in selection. Procedural justice is one of the most influential determinants of perceived fairness of selection tools and it can be argued that these methods are acceptable to stakeholders because they are viewed as procedurally just. Prior research has shown that the extent to which a selection tool is viewed as job related exerts the greatest influence on perceptions of procedural justice [9, 21]. This review establishes that MMIs are considered by applicants, selectors and students, as highly authentic with immediate relevance to clinical practice. SCs and SJTs represent high to medium fidelity assessments and the job relatedness of these methods is similarly highly rated by applicants and selectors.

Another aspect of procedural justice is the concept of “*voice*” [20,21]. “*Voice*” describes adequate opportunity for the applicant to perform, to make a case for themselves as well as sufficient time to do so [107]. The fact that applicants and selectors view MMIs, SCs and SJTs as providing adequate opportunity for candidates to demonstrate their ability and allows for differentiation between candidates is likely to be a key factor in acceptability. In addition MMIs, SJTs and SCs involve selectors directly in selection judgements and by extension this provides them with an opportunity for *voice* in selection decisions. By contrast selectors are somewhat removed from decisions made by aptitude tests which may contribute to relatively poorer ratings of this tool.

Aptitude tests generally receive mixed stakeholder acceptability. Underrepresented and minority medical school applicants view them as barriers, while other applicants and medical students question their fairness, face and predictive validity. The job relatedness of some of the item formats is questioned in particular abstract reasoning test items, such as non-verbal reasoning questions. This reflects the experience outside of medicine with similar tools and it is recommended that one way to incorporate procedural justice into the design of cognitive tests is to use comparatively concrete item types [108].

Of concern to students and selectors is the perception that aptitude tests may be susceptible to coaching, and the associated fear that this may lead to economic bias. Concerns regarding the possibility that commercial coaching could lead to unfair advantage represent a breach of the distributive justice principle of equal opportunity. Research has shown that justice rules can be more influential, and weigh more heavily on overall estimation of acceptability, when they are violated rather than when they are satisfied [21, 24]. In practical terms, this could mean that even in a situation where a selection tool is considered to perform well across a number of other justice domains – it may still prove unacceptable to stakeholders if it is perceived to fall short in one regard.

There were very few records that explored stakeholder views of other methods such as letters of recommendation, essays and personal statements, but those that did expressed some reservation about the veracity of content. The predictive validity of these methods is also known to be limited, and this coupled with poor stakeholder acceptability, challenges their role in the selection process) [6, 109].

### Study limitations

One of the biggest limitations of this review is that the overall quality of the evidence was low based on the average MERSQI score. The low MERSQI scores are principally due to the majority of studies being conducted in single institutions, with single groups of stakeholders surveyed once, often immediately after exposure to one selection tool in a new or pilot setting, with limited evidence for the validity of the evaluation instrument. Furthermore due to the heterogeneity of study designs, a formal assessment of risk of bias that may affect the cumulative evidence (e.g. attrition bias, reporting bias, publication bias) was not performed. For both of these reasons, readers are advised to note that the quality of evidence in this review is relatively low and there may be potential for bias within and across studies.

A second major limitation of this review relates to the heterogeneity of the selection methods themselves. For instance not all MMIs are the same, in fact no two MMIs are the same [110]. Similarly there are important differences between aptitude tests, such as the degree to which they are designed to measure crystallised versus fluid intelligence [6]. A systematic review such as this one, that seeks to summarise and generalise overall stakeholders’ views, will inevitably mask these important contextual differences.

This review emphasises gaps and shortcomings in the research evidence of stakeholder views of selection to medicine. For example, no studies were identified during the time frame of this review, exploring participants’ views of personality assessments and only scant exploration of academic record which confirms that there are gaps in our understanding of stakeholder views. However the authors acknowledge that the time frame of this review may have excluded research on some of the longer established methods, such as academic record.

With respect to methodology, this review revealed a predominance of quantitative research. Qualitative research, on the other hand, is ideally suited to understanding the meaning of selection for the respective stakeholder groups and can greatly add to our understanding of the views and attitudes of stakeholders. The use of theoretical models, to conceptualise and interpret stakeholder views, was rare in this review, but again can help us to better appreciate and compare the nuances of stakeholder acceptability.

Within the quantitative paradigm, the use of standardised methods would better facilitate higher standards of reporting of the content and internal validity of the evaluation instrument, and would accommodate comparisons between different stakeholder evaluations. Transparently transmitting this information in an interpretable manner to stakeholders should assume more importance. Equally, there is a need for better prioritisation of stakeholder views as a legitimate aim for selection research. Future research should consider this, given the centrality of stakeholder views to the political validity of selection methods and the potential to which negative perceptions can deter already marginalised applicants and negatively influence their opinion of the medical professions.

In addition future research should aim to follow up the views of unsuccessful applicants and to seek the views of a wider pool of stakeholders. For example, no studies exploring the views of medical school applicants’ parents were found, yet they are likely to be substantially invested in the application process. Also, there was limited research on the views of patients, or general public or members of the medical profession outside of those directly involved in the admission process or clinical teaching. Similarly, there was only one study of career guidance officers identified yet this group has been noted to be potentially very influential on applicants’ preparation for medical school admission [44, 81, 90]. Finally, while there have been many studies of stakeholders’ views, for the most part, each group is treated as if it is homogenous. Future research should be mindful of these issues and seek to sensitively explore views in a manner that accommodates both differences and similarities within stakeholder groups.

## Conclusions

Stakeholders in medical student selection are a collection of diverse groups with potentially differing views. It is critical to the operation of fair and defensible selection processes that we understand and appreciate the range and depth of views that they hold. It is incumbent upon all involved in the selection process to ensure that accurate information is available to all stakeholders and that there is clarity regarding the objectives and purpose of each selection method used to allocate a place in medicine. This review demonstrates that there is important work being done in this field, especially in respect to applicants. However, it highlights the need for better standards and more appropriate methodologies; for broadening the scope of the stakeholder groups included in future research. Finally we hope this review reinforces recognition that stakeholders even from the same group are not necessarily homogenous. Their perceptions are significantly influenced by a range of cultural and environmental factors as well as information disseminated by those responsible for selection.

## Additional files

Additional file 1: Example of search using “Stakeholder Views” search strategy on Ovid Medline. (DOCX 16 kb)

Additional file 2: The completed MERSQI checklist for each included record, tabulated. (DOCX 32 kb)

## Abbreviations

CPST: Clinical problem-solving test
GAMSAT: Graduate medical school admissions test
GP(s): General practitioner(s)
GPA: Grade point average
HPAT-Ireland: Health professions admission test-Ireland
IMGs: International medical graduates
LOR: Letters of recommendation
MCAT: Medical college admission test
MMI(s): Multiple mini interview(s)
SJT: Situational judgement test
TI: Traditional interview
UCAS: The universities and colleges admissions service
UKCAT: UK clinical aptitude test
UMAT: Undergraduate medicine and health sciences admission test
URM: Under-represented minorities

## Glossary

Distributive justice: Refers to the perceived fairness of organisational outcome distributions (Chambers, 2002).
Interactional justice: Describes the interpersonal treatment people receive as organisational procedures are enacted (Chambers, 2002).
Organisational justice theories: Describe perceptions of fairness in organisational processes (Colquitt et al., 2005).
Procedural justice: Relates to the perceived fairness of the procedures used to arrive at the resource allocation decisions (Chambers, 2002).
Stakeholder: Those who are affected by or can affect selection processes (adapted from Freeman, 2010).
