# Correction to: A systematic review of stakeholder views of selection methods for medical schools admission

**DOI:** 10.1186/s12909-018-1271-6

**Published:** 2018-07-05

**Authors:** M. E. Kelly, F. Patterson, S. O’Flynn, J. Mulligan, A. W. Murphy

**Affiliations:** 1 Discipline of General Practice, Clinical Science Institute, National University of Ireland, Galway, Ireland; 2 University College Cork, Cork, Ireland; 3 Work Psychology Group, Derby, UK

## Correction

Following publication of the original article [1], the author report typographical errors in the

Figure 1 Study Search Strategy and Review Process – the lowest box on the right hand side should read “Full-texts excluded with reasons (n=37)” instead of “(n=38)”

Page 3 of the text, column 2, paragraph 2, second-last sentence should be “Subsequently 71 records were included for full review and 37 excluded” instead of “Subsequently 70 records were included for full review and 38 excluded”
