# Supplementary material for: A systematic review of stakeholder views of selection methods for medical schools admission
Source: BMC Med Educ. 2018 Jun 15;18:139. doi: 10.1186/s12909-018-1235-x (PMC6002997; doi:10.1186/s12909-018-1235-x)
Supplement: Supplementary file 1 — Example of search using “Stakeholder Views” search strategy on Ovid Medline. (DOCX 16 kb) [file 12909_2018_1235_MOESM1_ESM.docx]

#### Stakeholder Views Search Strategy Ovid Medline

| **Searches** | **Results** | **Search Type** |
| --- | --- | --- |
| **41** | **limit 40 to yr="2000 - 2014"** | **268** |
| 40 | 18 and 25 and 31 and 39 | 393 |
| 39 | 32 or 33 or 34 or 35 or 36 or 37 or 38 | 17243 |
| 38 | ("residency selection$" or "select$ to residency" or "select$ for residency" or "recruit$ to residency" or "recruit$ for residency" or "training selection" or "select$ to training" or "select$ for training" or "training recruit$" or "recruit$ to training" or "recruit$ for training").mp. [mp=title, abstract, original title, name of substance word, subject heading word, keyword heading word, protocol supplementary concept word, rare disease supplementary concept word, unique identifier] | 1609 |
| 37 | ("medical school$ admission" or "admission$ polic$" or "medical school$ entr$").mp. [mp=title, abstract, original title, name of substance word, subject heading word, keyword heading word, protocol supplementary concept word, rare disease supplementary concept word, unique identifier] | 520 |
| 36 | ("medical student$ selection” or select$ medical student$*" or "medical student$ admission$" or "admit$ medical student$").mp. [mp=title, abstract, original title, name of substance word, subject heading word, keyword heading word, protocol supplementary concept word, rare disease supplementary concept word, unique identifier] | 17 |
| 35 | exp Personnel Selection/ | 11107 |
| 34 | Entrance examinations.mp. | 58 |
| 33 | exp College Admission Test/ | 499 |
| 32 | exp School Admission Criteria/ | 4244 |
| 31 | 26 or 27 or 28 or 29 or 30 | 145000 |
| 30 | exp "Internship and Residency"/ | 36732 |
| 29 | exp Education, Medical, Graduate/ | 22420 |
| 28 | exp Education, Medical, Undergraduate/ | 18619 |
| 27 | exp Education, Medical/ | 132711 |
| 26 | exp Schools, Medical/ | 21241 |
| 25 | 19 or 20 or 21 or 22 or 23 or 24 | 942374 |
| 24 | (view* or opinion* or perspective* or belie* or attitude*or accept*).mp. [mp=title, abstract, original title, name of substance word, subject heading word, keyword heading word, protocol supplementary concept word, rare disease supplementary concept word, unique identifier] | 707830 |
| 23 | student attitude.mp. | 44 |
| 22 | job applicant attitude.mp. | 0 |
| 21 | exp Public Opinion/ | 15867 |
| 20 | exp "Attitude of Health Personnel"/ | 124030 |
| 19 | exp Attitude/ | 273753 |
| 18 | 1 or 2 or 3 or 4 or 5 or 6 or 7 or 8 or 9 or 10 or 11 or 12 or 13 or 14 or 15 or 16 or 17 | 616894 |
| 17 | public.mp. | 355221 |
| 16 | "school leaver*".mp. | 235 |
| 15 | applicant*.mp. | 5507 |
| 14 | stakeholder*.mp. | 13274 |
| 13 | "Overseas student*".mp. | 67 |
| 12 | "international student*".mp. | 279 |
| 11 | "international medical graduate*".mp. | 528 |
| 10 | exp Patients/ | 69769 |
| 9 | exp Physicians, Primary Care/ | 1475 |
| 8 | exp Foreign Medical Graduates/ | 3058 |
| 7 | exp General Surgery/ | 34841 |
| 6 | exp General Practitioners/ | 2350 |
| 5 | exp Physicians/ | 89482 |
| 4 | exp Medical Staff/ | 24324 |
| 3 | exp "Internship and Residency"/ | 36732 |
| 2 | exp Faculty, Medical/ | 10248 |
| 1 | exp Students, Medical/ | 22673 |
