# Supplementary material for: A systematic review of stakeholder views of selection methods for medical schools admission
Source: BMC Med Educ. 2018 Jun 15;18:139. doi: 10.1186/s12909-018-1235-x (PMC6002997; doi:10.1186/s12909-018-1235-x)
Supplement: Supplementary file 2 — The completed MERSQI checklist for each included record, tabulated. (DOCX 32 kb) [file 12909_2018_1235_MOESM2_ESM.docx]

Additional File 2 Completed MERSQI Checklist for Assessing the Quality of Included Studies

| *Study* | *Study Design (max 3)* | *Sampling (max 3)* | *Type of data (max 3)* | *Validity of evaluation instrument (max 3)* | *Data Analysis*  *(max 3)* | *Outcomes (max 3)* | *Overall Score*  *(max 18)* |
| --- | --- | --- | --- | --- | --- | --- | --- |
| Adams 2009 [52] | 1 | 1.5+0.5 | 1 | 0+0+0 | 1+1 | 1 | 7 |
| Agrawal et al 2005 [53] | 1 | 1.5 +1 | 1 | 1+0+0 | 1+1 | 1 | 8.5 |
| Brown & Griffin 2012 [54] | 2 | 0.5+0.5 | 1 | 0+1+0 | UTD+UTD | 1 | 6 |
| Brownell et al 2007 [55] | 2 | 0.5+1.5 | 1 | 0+0+0 | 1+1 | 1 | 8 |
| Campagna-Vaillancourtet al 2013 [45] | 2 | 1+1.5 | 1 | 0+0+0 | 1+1 | 1 | 8.5^ |
| Christakis et al 2010 [56] | 2 | 1+1.5 | 1 | 0+0+0 | 1+1 | 1 | 7.5 |
| Cleland et al 2011 [46] | 1 | 1.5+1.5 | 1 | 0+0+0 | 1+2 | 1 | 9^ |
| Daram et al 2014 [57] | 1 | 0.5+0.5 | 1 | 0+0+0 | 1+1 | 1 | 6 |
| Dennehy et al 2013 [58] | 1 | 1+1.5 | 1 | 0+0+0 | 1+1 | 1 | 7.5 |
| Dhar et al 2012 [59] | 1 | 1+1 | 1 | 0+0+0 | 1+2 | 1 | 8 |
| Dore et al 2010 [60] | 2 | 1+0.5 | 1 | 1+0+0 | 1 | 1 | 7.5 |
| Dowell et al 2012 [61] | 2 | 0.5+1 | 1 | 0+0+0 | 1+1 | 1 | 7.5 |
| El Says et al 2013 [ 62] | 1 | 0.5+0.5 | UTD | UTD | UTD | 1 | 3 |
| Eva et al 2004 [ 63] | 2 | 0.5+0.5 | 1 | 0+0+0 | 1+1 | 1 | 7 |
| Eva et al 2004 [ 64] | 2 | 0.5+0.5 | 1 | 0+0+0 | 1+1 | 1 | 7 |
| Eva & Macala 2014 [ 65] | 2 | 0.5+0.5 | 1 | 0+0+0 | 1+2 | 1 | 8 |
| Gale et al 2010 [ 66] | 2 | 0.5+0.5 | 1 | 0+0+0 | 1+1 | 1 | 7 |
| Goulston & Oates 2009 [ 47] | 2 | 0.5+0.5 | 1 | 0+0+0 | UTD | UTD | 4 |
| Griffin et al 2008 [67 ] | 1 | 1+1.5 | 1 | 0+0+0 | 1+1 | 2 | 8.5 |
| Gula 2014 [68 ] | 2 | 1+0.5 | 1 | 0+0+0 | UTD +UTD | UTD | 4.5 |
| Harris & Owen 2007 [ 69]  **Key:** ***NR= not relevant because qualitative study, UTD –unable to determine, ^indicates mixed methods studies with the MERSQI score applied to the quantitative strand.*** | 1 | 0.5+0.5 | 1 | 0+1+0 | 1+1 | 1 | 7^ |
| Henry 2006 [ 70] | 1 | 0.5+0.5 | 1 | 1+1+0 | 1+1 | 1 | 8 |
| Hofmeister et al 2008 [48 ] | 2 | 1+1.5 | 1 | 0+0+0 | 1+1 | 1 | 8.5^ |
| Hopson et al 2014 [ 71] | 1.5 | 1.5+1.5 | 1 | 0+0+0 | 1+2 | 1 | 10 |
| Humphrey et al 2008 [72 ] | 2 | 1+1.5 | 1 | 0+1+0 | 1+1 | 1 | 9.5 |
| Husbands et al 2014 [73 ] | 1 | UTD+ 0.5 | 1 | 0+0+0 | UTD+UTD | 1 | 3.5 |
| Jauhar et al 2008 [74 ] | 1 | 1.5+1 | 1 | 1+0+0 | 1+1 | 1 | 8.5 |
| Jayasuriya et al 2012 [37 ] | NR | NR | NR | NR | NR | NR | NR |
| Johnson & Elam 2001 [ 75] | 2 | 0.5+1.5 | 1 | 0+0+0 | 1+1 | 1 | 8 |
| Kaffenberger et al 2014 [76 ] | 1 | 1+0.5 | 1 | 0+0+0 | 1+1 | 1 | 6.5 |
| Kelly et al 2014 [77 ] | 2 | 0.5+1 | 1 | 0+0+0 | 1+1 | 1 | 8 |
| Kelly et al 2014 [38] | NR | NR | NR | NR | NR | NR | NR |
| Kleshinski et al 2008 [78] | 2 | 0.5+1 | 1 | 0+0+0 | 1+1 | 1 | 7.5 |
| Koczwara et al 2012 [79 ] | 1 | 0.5+1.5 | 1 | 1+0+0 | 1+1 | 1 | 8 |
| Kumar et al 2009 [39 ] | NR | NR | NR | NR | NR | NR | NR |
| Kumwenda et al 2013 [80 ] | 1 | 1.5+0.5 | 1 | 1+1+0 | 1+1 | 1 | 9 |
| Lambe et al 2012 [81 ] | 1 | 0.5 +1 | 1 | 0+0+0 | 1+1 | 1 | 6.5 |
| Lievens & Sackett 2006 [ 83] | 2 | 1.5+1 | 1 | 1+1+0 | 1+1 | 1 | 10.5 |
| Lievens 2013 [82 ] | 1 | 1.5+1 | 1 | 1+0+0 | 1+1 | 1 | 8.5 |
| Lubarsky &Young 2013 [ 84] | 1 | 0.5+0.5 | 1 | 0+0+0 | UTD +UTD | 1 | 4 |
| Marrin et al 2004 [49 ] | 2 | 0.5+1 | 1 | 0+1+0 | 1+1 | 1 | 8.5 |
| Mathers & Parry 2010 [40 ] | NR | NR | NR | NR | NR | NR | NR |
| Milne et al 2001 [85 ] | 1 | 0.5+1.5 | 1 | 1+0+0 | 1+1 | 1 | 8 |
| Mitchson 2009 [86] | 1 | 0.5+1.5 | 1 | 0+0+0 | 1+1 | 1 | 7 |
| Monroe et al 2013 [87 ] | 1 | 1.5+1.5 | 1 | 0+1+0 | 1+1 | 2 | 10 |
| Niyomdecha et al 2012 [88 ] | 1 | 0.5+0.5 | 1 | 0+0+0 | UTD+UTD | 1  **Key:** ***NR= not relevant because qualitative study, UTD –unable to determine, ^indicates mixed methods studies with the MERSQI score applied to the quantitative strand.*** | 4 |
| O'Brien et al 2011 [ 89] | 2 | 0.5+0.5 | 1 | 0+0+0 | 1+1 | 1 | 7 |
| O’Flynn et al 2013 [90 ] | 1 | 1.5+0.5 | 1 | 0+0+0 | 1+1 | 1 | 7 |
| Patel et al 2011 [ 91] | 1 | 0.5+0.5 | 1 | 0+0+0 | 1+1 | 1 | 6 |
| Patterson et al 2009 [ 92] | 2 | 0.5+0.5 | 1 | 1+0+0 | 1+1 | 1 | 8 |
| Patterson et al 2011 [7 ] | 1 | 1.5+1 | 1 | 1+1+0 | 1+1 | 1 | 9.5 |
| Patterson et al 2013 [93 ] | 1 | UTD | 1 | 0+0+0 | UTD | 1 | 3 |
| Randall et al 2006 [94 ] | 1 | 0.5+0.5 | 1 | 0+0+0 | 1+1 | 1 | 6 |
| Razack et al 2009 [50 ] | 2 | 0.5+1.5 | 1 | 0+0+0 | 1+1 | 1 | 8^ |
| Rich 2011 [95 ] | 2 | 0.5+0.5 | 1 | 0+0+0 | UTD +UTD | 1 | 5 |
| Rodgerson et al 2013 [ 96] | 1 | 0.5+1 | 1 | 0+0+0 | UTD+UTD | 1 | 4.5 |
| Samarasekera et al 2014 [ 97] | 2 | 0.5+0.5 | 1 | 0+0+0 | UTD+UTD | 1 | 5 |
| Stagg & Rosenthal 2012 [41 ] | NR | NR | NR | NR | NR | NR | NR |
| Stevens et al 2013 [51 ] | 1 | 1.5+1.5 | 1 | 0+0+0 | 1+1 | 1 | 8^ |
| Tiller et al 2013 [98 ] | 2 | 1+1 | 1 | 0+0+0 | 1+1 | 1 | 8 |
| Turner & Nicholson 2011 [ 42] | NR | NR | NR | NR | NR | NR | NR |
| Uijtdehaage et al 2011 [ 99] | 2 | 0.5+1.5 | 1 | 1+0+0 | 1+1 | 1 | 9 |
| UKCAT Consortium 2009/2010 [100] | 1 | 1.5+0.5 | 1 | 0+0+0 | 1+1 | 1 | 6 |
| UKCAT Consortium 2011 [ 101] | 1 | 1.5+0.5 | 1 | 0+0+0 | 1+1 | 1 | 6 |
| Vermeulen et al 2012 [ 102] | 1 | UTD+0.5 | 1 | 0+0+0 | UTD+1 | 1 | 4.5 |
| Waheed et al 2011 [103] | 2 | 0.5+ 0.5 | 1 | 0+0+0 | 1+1 | 1 | 7 |
| Westwood et al 2008 [104] | 1 | 0.5+1.5 | 1 | 0+0+0 | 1+1 | 1 | 7 |
| White et al 2011 [43 ] | NR | NR | NR | NR | NR | NR | NR |
| Wilkinson & Wilkinson 20 13 [105] | 2 | 0.5+0.5 | 3 | 0+0+0 | 1+2 | 1.5 | 10.5 |
| Wright 2012 [44 ] | NR | NR | NR | NR | NR | NR | NR |
| Ziv et al 2008 [106 ] | 2 | 0.5+0.5 | 1 | 0+0+0 | 1+1 | 1 | 7 |

**Key:** ***NR= not relevant because qualitative study, UTD –unable to determine, ^indicates mixed methods studies with the MERSQI score applied to the quantitative strand.***
